# A novel polysaccharide from *Sargassum integerrimum* induces apoptosis in A549 cells and prevents angiogensis *in vitro* and *in vivo*

**DOI:** 10.1038/srep26722

**Published:** 2016-05-24

**Authors:** Ge Liu, Shan Kuang, Shimei Wu, Weihua Jin, Chaomin Sun

**Affiliations:** 1 Key Laboratory of Experimental Marine Biology, Institute of Oceanology, Chinese Academy of Sciences, Qingdao, 266071, China; 2 Laboratory for Marine Biology and Biotechnology, Qingdao National Laboratory for Marine Science and Technology, Qingdao, 266071, China; 3 College of Earth Science, University of Chinese Academy of Sciences, Beijing, 100049, China; 4 Key Laboratory of Biobased Materials, Qingdao Institute of Bioenergy and Bioprocess Technology, Chinese Academy of Sciences, Qingdao, 266101, China

## Abstract

Many polysaccharides isolated from plants have exhibited promising antitumor activities. The aim of this study is to investigate the antitumor activity of the novel polysaccharide named SPS from *Sargassum integerrimum*, elucidate the underlying anticancer mechanism in a human lung cancer cell line A549, and evaluate its anti-angiogenic activity both *in vitro* and *in vivo*. The results show that SPS significantly reduces A549 cells viability in a dose- and time-dependent manner via MTT method. Flow cytometry analysis indicates that SPS could induce cell apoptosis, the loss of mitochondrial membrane potential (MMP), generation of reactive oxygen species (ROS) and G2/M phase cell cycle arrest of A549 cells. Up-regulation of the expressions of P53 and Bax, down-regulation of the expression of Bcl-2, and activation of cleaved caspase-3, caspase-9 and PARP are also detected by western blotting after the treatment of SPS. In addition, SPS inhibits the proliferation, migration and cord formation of human umbilical vein endothelial cells (HUVECs) *in vitro*, and prevents the vascular development of zebrafish embryos *in vivo*. Altogether, our data prove the anticancer and anti-angiogenesis properties of SPS, and provide further insights into the potential pharmacological application of SPS as antitumor and anti-angiogenic agent against lung cancer.

Lung cancer is the leading cause of cancer-related death in both man and woman all over the world, and the effectiveness of current treatment is severely limited, with an age-standardized mortality rate of 30.0 per 100,000^1^. Non-small cell lung cancer (NSCLC) accounts for about 80% of all types of lung cancer^2^ and chemotherapy is still the standard treatment method. Although the chemotherapy-based treatment has tremendously improved the symptoms and quality of life of patients with NSCLC, the overall survival rate still remains at a low level. Therefore, there is a highly urgent desire to develop novel and effective agents for the prevention and treatment of non-small cell lung cancer.

Apoptosis, also called type I programmed cell death, is a strictly regulated process of physiological cell death. It evokes cell death through intrinsic (via mitochondria) or extrinsic (via death receptors) pathways and plays an essential role in maintaining tissue homeostasis and normal cell proliferation. The defective apoptosis, together with the uncontrolled cell proliferation can disturb the balance between cell death and cell division, finally resulting in the cancer development^3^,^4^. Thereby, drugs that can promote apoptosis and inhibit dysfunctional cell proliferation may be effective to prevent the cancer progression^5^.

In addition, anti-angiogenesis therapies have become a hot spot in cancer treatment. Angiogenesis is the physiological process to form new blood vessels from pre-existing ones, which involves numerous coordinated endothelial cell activities, including proliferation, migration, alignment and cord formation^6^. It has been well established that angiogenesis plays an important role in tumor growth, transplantation and metastasis^7^, because a functional blood system is required to supply nutrients and oxygen, and to remove waste products^8^. Hence, anti-angiogenic therapy has become a promising and valid approach in the development of novel anticancer therapy^9^. In recent years, a large number of anti-angiogenic agents have progressed to be studied and thereafter used clinically^10^. However, most of these anti-angiogenic agents usually cause normal endothelial cell dysfunction, induce drug resistance of cancer, and do not target tumor cells specifically^11^. Therefore, there is a highly urgent need to search for safer and more effective anti-angiogenic agents for the treatment of cancer.

Recently, many polysaccharides from natural source have been found to possess a variety of biological activities, including antitumor and anti-angiogenic activities, and have attracted enormous attention in biochemical and medical areas^12^. Numerous studies have reported that natural polysaccharides can inhibit tumor cell proliferation either by directly inducing apoptosis or by triggering immunopotentiation activity in combination with chemotherapy^13^,^14^. Moreover, it is also well documented that some polysaccharides from seaweeds such as G*rateloupia longifolid*^15^, *Fucus vesiculosus*^16^ and *Codium cylindricum*^17^ displayed potent anti-angiogenic activity. More importantly, many of the natural polysaccharides are found to be effective and relatively nontoxic substances. Thus, natural polysaccharides possessing the splendid activities of inducing apoptosis and anti-angiogenesis might be ideal candidates for oncotherapy and adjuvant therapy.

Marine algae are rich sources of structurally diverse, biologically active compounds with great potential in the pharmaceutical, food, and biomedical industries^18^ and they can be mainly classified into three classes based on their pigmentation: Brown algae, Green algae, and Red algae. Among them, brown algae are known to have many different bioactive compounds, including polysaccharides and polyphenols^19^. In particular, *Sargassum*, a genus of brown seaweed, has been reported to produce polysaccharides with a low fucose and sulfate content, which possesses neuroprotective^20^, antioxidant^21^ and anticancer activities^22^.

In this study, we isolated and purified a novel polysaccharide SPS from *Sargassum integerrimum*, which is endemic to China and distributes mainly in Naozhou island area of Zhanjiang, Guangdong Province. Moreover, the anti-cancer activities of SPS against human non-small cell lung cancer cell line (A549) and the anti-angiogenic properties both *in vitro* and *in vivo* are also investigated. Our data show that SPS exerts the proliferation-inhibiting effect on A549 cells by inducing mitochondria-mediated apoptotic cell death and cell cycle arrest, and SPS prevents formation of angiogenesis of HUVECs and zebrafish embryos, indicating that SPS could be developed as a novel and potential anti-tumor and anti-angiogenic drug against lung cancer.

## Results

### SPS inhibited the proliferation of A549 cells

**Figure 1:**
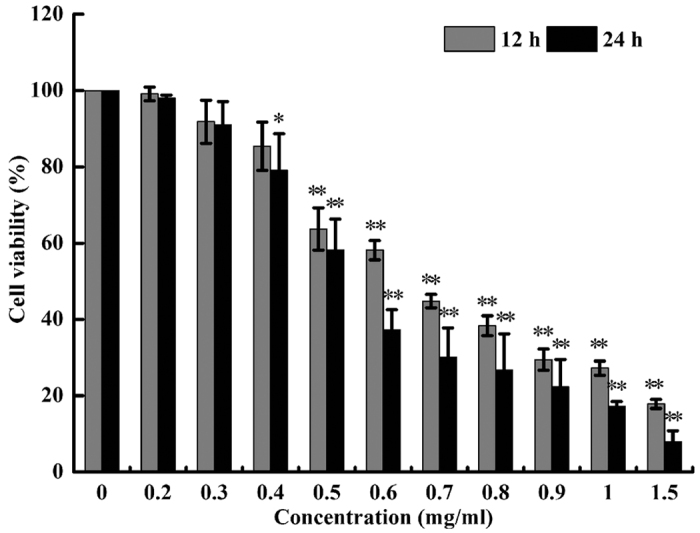
Concentration- and time-dependent cytotoxic effects of SPS on A549 cells. Cells were cultured in 96-well plate and treated with different doses of SPS (0–1.5 mg/ml) for 12 and 24 h. The cell viability was analyzed by MTT assay. Data are presented as means ± SD of three independent experiments (n = 3). *P < 0.05, **P < 0.01 *versus* medium control.

In order to evaluate the proliferation inhibition by SPS, A549 cells were exposed to increasing concentrations of SPS for 12 and 24 h, and cell viability was measured by MTT assay. As shown in Fig. 1, SPS markedly inhibited the growth of A549 cells in a time- and dose-dependent manner. After incubation for 24 h, the inhibition rate of SPS increased from about 2 to 92%, and the highest inhibitory rate was up to 92.1% when its concentration increased to 1.5 mg/ml. The IC_50_ values at 12 h and 24 h were calculated to be 0.67 mg/ml and 0.49 mg/ml, respectively.

### SPS induced apoptosis in A549 cells

**Figure 2:**
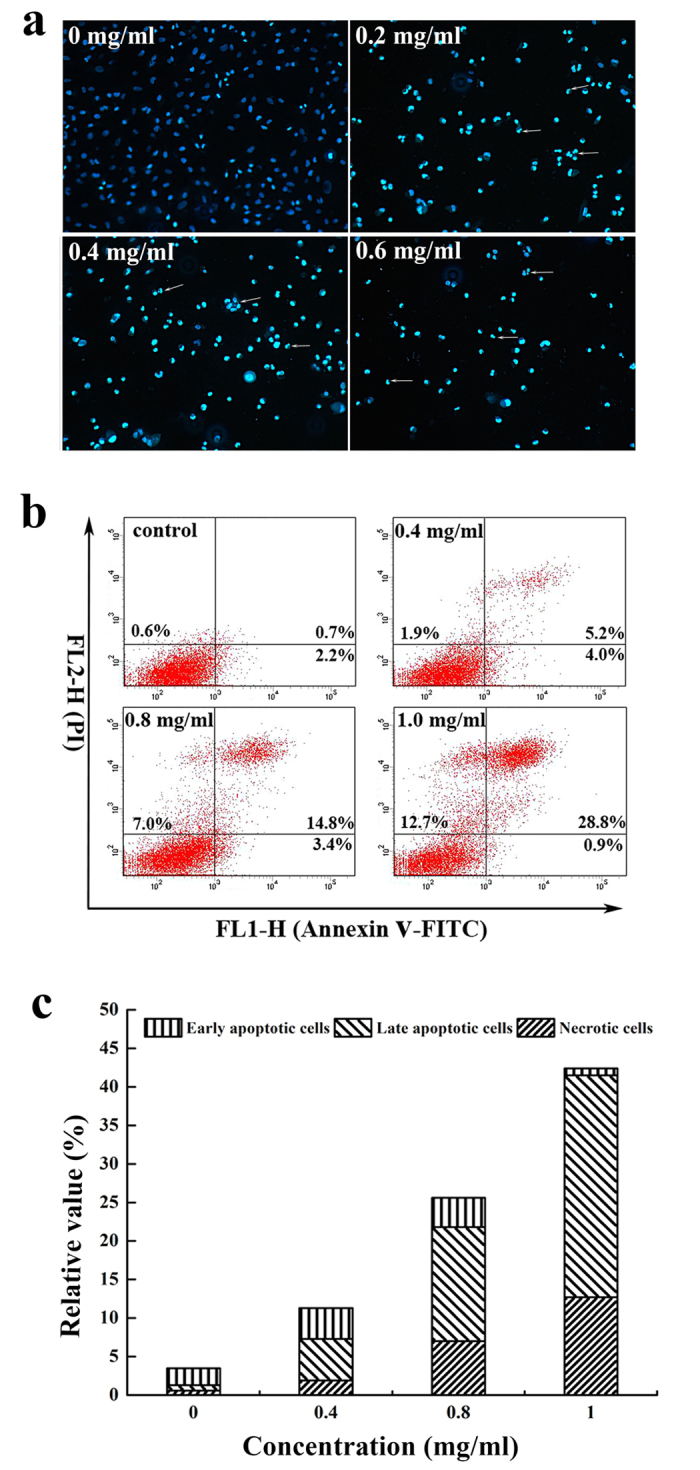
Effects of SPS on cell apoptosis in A549 cells. (**a**) After treated with 0, 0.2, 0.4 and 0.6 mg/ml SPS for 12 h, A549 cells were stained by Hoechst 33258 solution and visualized by a fluorescence microscopy. White arrows indicated the sublobe, fragment shape, and fringe collection of cell nucleus. (**b**) Representative dot plots of Annexin V/PI staining. A549 cells were treated with indicated concentrations of SPS (0, 0.4, 0.8 and 1.0 mg/ml) for 12 h, and stained with Annexin V-FITC/PI solutions according to the manufacturer’s manual, and detected using flow cytometry. (**c**) Column bar graph of apoptotic cells. Annexin V^−^/PI^−^ (lower left) cells were represented survivals, Annexin V^+^/PI^−^ (lower right) cells were defined as early apoptotic cells, Annexin V^+^/PI^+^ (upper right) cells were recognized as late apoptotic cells, Annexin V^+^/PI^−^ (upper left) cells were considered as necrotic apoptotic cells. All experiments were performed n = 3 in replicates.

In order to investigate whether the growth-inhibitory effect is related to the induction of apoptosis, A549 cells were treated with 0, 0.2, 0.4 and 0.6 mg/ml SPS for 12 h and the nuclear morphological changes of A549 cells were confirmed by Hoechst 33258 staining (Fig. 2a). Compared with the normal nuclear morphology of the control cells, the cells treated by SPS presented typical morphological characteristics of apoptosis, including nuclear pyknosis, sublobe, fragment shape, and fringe collection. Further confirmation of apoptosis induced by SPS was performed by flow cytometry based on Annexin V-FITC/PI double staining.

The results of flow cytometry analysis (Fig. 2b,c) showed that the apoptosis of A549 cells were remarkably induced after treated with SPS for 12 h, and treatment of A549 cells with SPS in concentrations of 0, 0.4, 0.8 and 1.0 mg/ml resulted in a dose-dependent increase in the numbers of late apoptotic and necrotic cells, from 0.7 to 28.8%, and 0.6 to 12.7%, respectively. These data suggested that the induction of apoptosis at least partly accounted for the growth inhibition of A549 cells.

### SPS induced the loss of mitochondrial membrane potential (MMP)

It is generally accepted that the process of apoptosis involves two pathways: the extrinsic pathway and intrinsic pathway, also called the death receptor pathway and mitochondrial pathway, respectively, and the molecular mechanisms involved have been well elucidated up to now. Mitochondrion has been shown to play an important role in the regulation of the intrinsic cell death^23^ and the dissipation of the mitochondrial membrane potential (MMP) activated by multiple stress signals is recognized as an irreversible step in the death cascade^24^. The loss of MMP is also thought to be an important event in the mitochondrial apoptotic pathway^25^.

**Figure 3:**
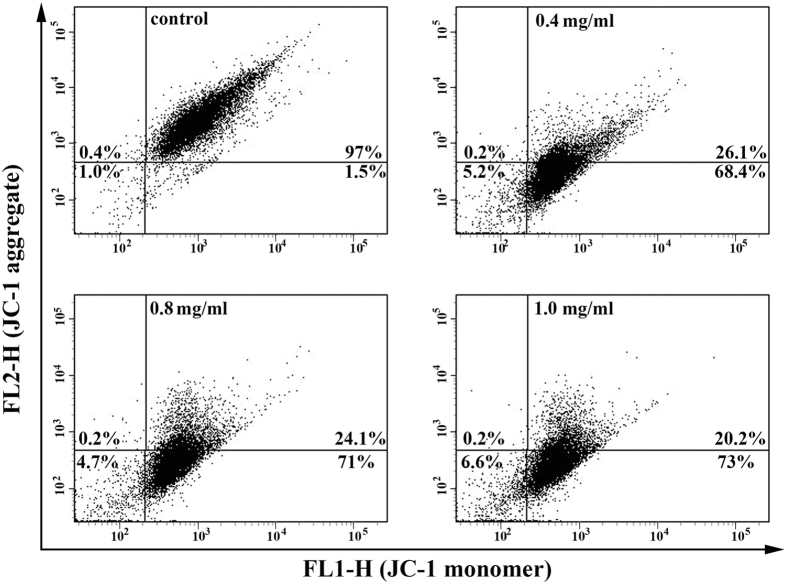
SPS induced the loss of mitochondrial membrane potential in A549 cells. After treated with different concentrations of SPS (0, 0.4, 0.8 and 1.0 mg/ml) for 12 h, cells were collected, washed, stained by JC-1, and then detected by flow cytometry. All assays were conducted in replicates.

To investigate the role of mitochondria in the apoptosis induced by SPS, the effect of SPS on MMP was measured by flow cytometry after A549 cells were stained with JC-1, which is capable of selectively entering mitochondria to form monomers that emit green fluorescence at low MMP, and form JC-1 aggregates that emit red fluorescence at high MMP. Compared with the control group, the number of treated cells emitting red fluorescence significantly decreased while the number of treated cells emitting green fluorescence obviously increased after treated for 12 h with SPS, which suggested the disruption of MMP (Fig. 3). These data indicated that the dissipation of MMP might participate in apoptosis induced by SPS.

### SPS induced the generation of reactive oxygen species (ROS)

Oxidative stress refers to an imbalance between pro-oxidant and anti-oxidant factors, and such imbalances may lead to cellular damage^26^. Reactive oxygen species (ROS) including superoxide, hydroxyl radical, hydrogen peroxide and singlet oxygen, are the byproducts of mitochondrial respiration chain, and play a key role in oxidative stress. Once ROS accumulate, they can attack many cellular components, such as nucleic acids, proteins and membrane lipids, and finally lead to cell death. Mitochondria are recognized as the predominate source of cellar ROS^27^, and the generation of cellular ROS is often associated with the loss of MMP^28^. Excessive intracellular ROS destroyed the mitochondrial membrane integrity, leading to Cytochrome *c* release, caspase activation, and finally apoptosis^29^.

**Figure 4:**
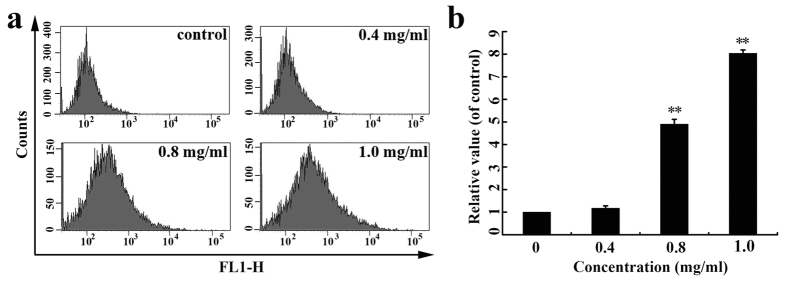
SPS induced the generation of reactive oxygen species (ROS) in A549 cells. (**a**) Cells were treated with the indicated concentrations of SPS (0, 0.4, 0.8 and 1.0 mg/ml) for 12 h and stained with DCHF-DA, followed by analyzed by flow cytometry. (**b**) Quantitative evaluations of ROS production triggered by SPS. Data are presented as a percentage relative to the control group (**P < 0.01 versus control group). All experiments were performed n = 3 in replicates.

Since we tested that SPS could cause the collapse of MMP, we next examined the intracellular ROS generation induced by SPS in A549 cells using DCFH-DA staining. As shown in Fig. 4a, the fluorescence intensity significantly increased compared with the control cells after treated with SPS for 12 h and the generation of ROS triggered by SPS was in a concentration-dependent manner (Fig. 4b). Therefore, we concluded that SPS could boost the production of ROS, which implied that SPS was an apoptotic stimulus and associated with the mitochondria-mediated apoptosis.

### Effects of SPS on proteins expressions related to apoptosis

**Figure 5:**
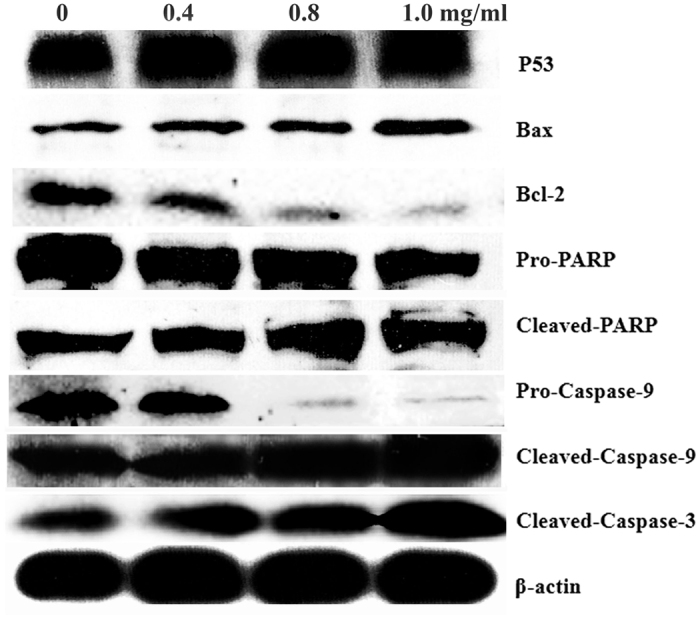
Effects of SPS treatment on the expression of proteins related to apoptosis by western blot analysis. A549 cells were treated with 0, 0.4, 0.8 and 1.0 mg/ml SPS for 24 h, and the expression levels of protein related to apoptosis were examined by western blot.

Apoptosis is a strictly regulated process, involving differences of a distinct protein expression. Among various factors related to apoptosis, some pro- and anti-apoptotic proteins can be used as markers of apoptosis. The tumor suppressor protein P53 works as both the inducer^30^ and regulator of apoptosis, and it has been proved to be involved in the control of DNA damage repair, cell cycle and apoptosis^31^. In this study, we conducted western blot analysis to examine the expression level of P53 in A549 cells and the results showed that the expression of P53 increased in a concentration-dependent manner after the cells were treated with SPS for 12 h (Fig. 5), indicating that the SPS-induced apoptosis in A549 cells was involved in the increasing expression of P53.

More and more evidence have demonstrated that Bcl-2 family proteins are the important mediators of apoptosis^32^ and play important roles in mitochondrial apoptotic pathway. Bcl-2 is an anti-apoptotic member of Bcl-2 family which prevented the cytochrome *c* release from mitochondria to cytosol, whereas Bax is a crucial pro-apoptotic member of Bcl-2 family which promoted the cytochrome *c* release from mitochondria to cytosol^33^. The ratio of Bax/Bcl-2 is a major checkpoint in the intrinsic apoptosis pathway^34^. Using western blot analysis, we found that, compared with the control group, the expression of Bcl-2 was down-regulated, but the expression of Bax was up-regulated after cells were treated for 12 h by SPS, resulting in an increase in the ratio of Bax/Bcl-2 in a dose-dependent manner (Fig. 5). The findings suggested that SPS treatment caused mitochondria-dependent apoptotic pathway in A549 cells through the up-regulation of Bax/Bcl-2 ratio.

Once cytochrome *c* is released from mitochondria into cytosol, it will combine with Apaf-1, ATP, and procaspase-9 to form the apoptosome assembly^35^ and trigger the activation of the caspase-dependent mitochondrial apoptotic pathway^36^. The apoptosome complex can activate initiator caspase-9, which then activates the downstream effector caspase-3 and induces subsequent cleavage of PARP to execute the apoptotic process^37^. To confirm the mechanism of SPS-induced apoptosis in A549 cells, we investigated the involvement of the mitochondria-mediated intrinsic apoptotic pathway by assessing the cleavage of caspases-3, caspase-9 and PARP by western blot analysis. As shown in Fig. 5, the expressions of the inactive form of caspase-9 and PARP decreased while the expressions of the cleaved caspase-3, caspase-9 and PARP increased after A549 cells were treated with SPS for 12 h, suggesting that mitochondria-mediated apoptotic pathway might be involved in SPS-induced apoptosis in A549 cells.

### SPS induced G2/M phase cell cycle arrest in A549 cells

**Figure 6:**
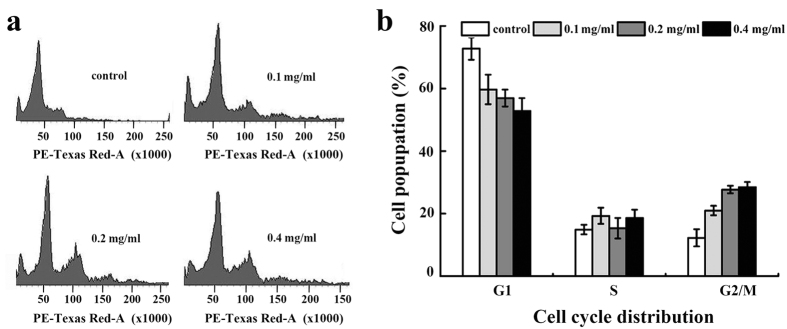
SPS induced G2/M phase cell cycle arrest in A549 cells. (**a**) Representative histograms of DNA content in the cells incubated with SPS at 0, 0.1, 0.2 and 0.4 mg/ml for 12 h. (**b**) Percentage of cell population in G1, S, and G2/M phase. All experiments were performed n = 3 in replicates.

To determine whether the growth inhibitory effect of SPS was due to cell cycle arrest, we investigated cell cycle phase distribution of A549 cells after treated with SPS for 12 h by flow cytometry. As shown in Fig. 6, with the increasing concentrations of SPS, the percentage of cells in G1 period reduced from 72.8 to 52.8%, while the proportion of cells in G2/M phrase increased from 12.3 to 28.5%, indicating that SPS predominantly induced G2/M phrase cell cycle arrest on A549 cells in a dose-dependent manner and partly contributed to the proliferation inhibition of A549 cells.

### SPS inhibited the proliferation of HUVECs

**Figure 7:**
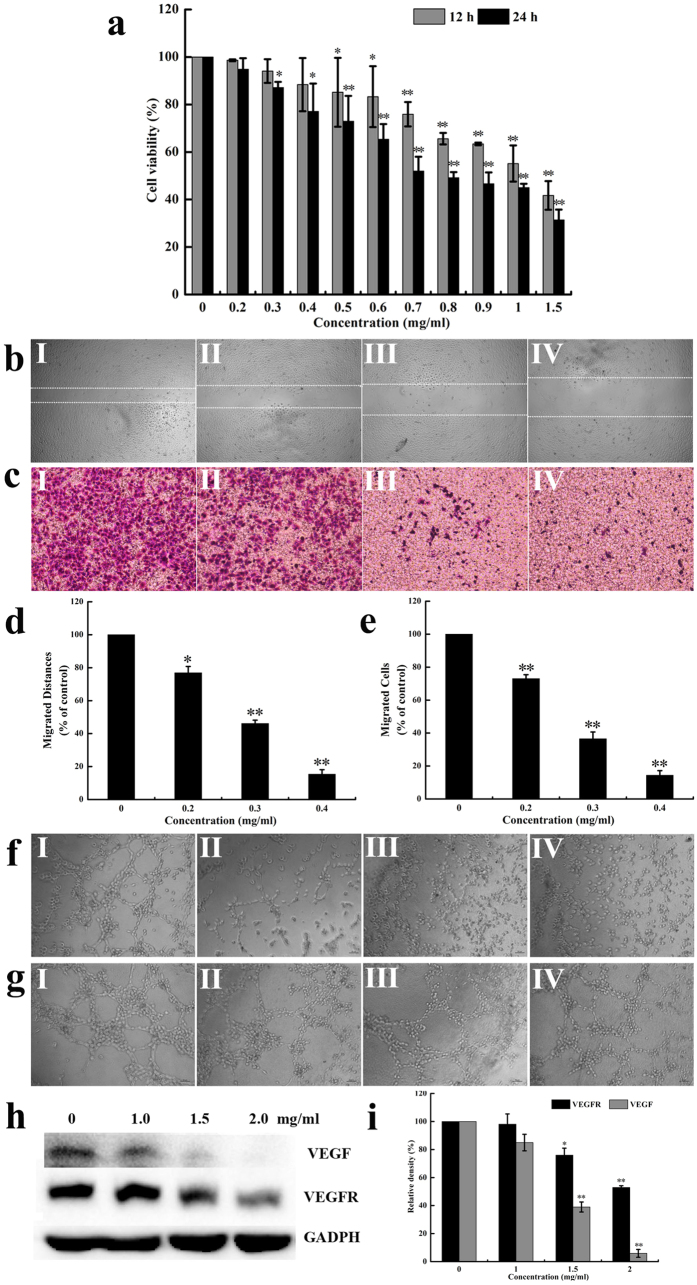
The anti-angiogenic activity of SPS on HUVECs *in vitro*. (**a**) Dose- and time-dependent growth-inhibiting effects of SPS on HUVECs. Cells were cultured in 96-well plate and treated with different concentrations of SPS (0–1.5 mg/ml) for 12 and 24 h. The cell viability was analyzed by MTT assay. Each data indicated the mean ± SD of three independent experiments (n = 3). *P < 0.05, **P < 0.01 *versus* medium control. After A549 cells were treated without (I) or with 0.2 (II), 0.3 (III) and 0.4 (IV) mg/ml of SPS for 8–10 h, cell migration was analyzed using scratch-wound assay (**b**) as well as Transwell assay (**c**). Quantitative evaluations of HUVECs migration induced by SPS in the scratch-wound assay (**d**) and Transwell assay (**e**). (**f**) SPS inhibited the formation of new capillary tube of HUVECs. HUVECs were plated on the surface of the Matrigel in a 96-well plate, where endothelial cells could align and form cords, and treated without (I) or with 0.2 (II), 0.3 (III) and 0.4 (IV) mg/ml of SPS for 6 h. (**g**) SPS did not disrupt the preformed vascular network of HUVECs. After the new tube-like structures established, various concentrations of SPS, 0 (I), 0.2 (II), 0.4 (III), and 0.6 (IV) mg/ml were added, and incubated for another 12 h. The disruption on the preformed tubes was observed and recorded using an inverted microscope. (**h**) After treated with SPS for 24 h, HUVECs were lysed and western blotting analysis was performed to detect the expressions of VEGF and VEGFR. (**i**) Histograms showed the quantitative evaluation of VEGF and VEGFR protein levels, which were measured by Image J and results were normalized to untreated cells.

Since endothelial cell proliferation is a key component of the angiogenic process, we checked the effect of SPS on the mitogenesis of HUVECs. After HUVECs were exposed to serial concentrations of SPS for 12 and 24 h, cell viability was measured using MTT method. The results showed that SPS exhibited a significant growth-inhibiting effect on HUVECs in a time- and dose-dependent manner (Fig. 7a). Compared with HUVECs without SPS treatment, the proliferation rate decreased by 44.9 and 58.3% after incubation for 12 h, and decreased by 55.0 and 68.6% after incubation for 24 h, when HUVECs were treated with SPS at concentrations of 1.0 and 1.5 mg/ml, respectively.

In addition, the cell cycle distribution of HUVECs treated with SPS was also detected to determine whether cell cycle arrest is related to the SPS-induced proliferation inhibitory effect. As shown in supplementary Fig. S1, the percentage of untreated cells in G2/M phase is 5.99%. However, when treated with 1.0, 1.5 and 2.0 mg/ml of SPS for 24 h, the G2/M phase proportion of cells increased to 10.35, 10.58 and 19.46%, respectively, indicating that SPS predominantly induced G2/M phase cell cycle arrest on HUVECs concentration-dependently and contributed to the proliferation inhibition of HUVECs.

### SPS repressed the migration of HUVECs

Migration of endothelial cells plays a vital role in angiogenesis, which is a chemical chemotaxis process. To investigate the influence of SPS on HUVECs migration, both scratch-wound assay and Transwell assay were employed in this study. The results of scratch-wound assay showed that SPS reduced the wound healing of HUVECs clearly (Fig. 7b), and the inhibition rate was in a dose-dependent relationship (Fig. 7d). In the Transwell assay, the data revealed that SPS treatment led to a concentration-dependent reduction in the number of migrated cells (Fig. 7c), and the inhibition rate of migrated cells was 42.9, 61.1, and 67.9%, when treated with 0.2, 0.3 and 0.4 mg/ml of SPS for 8 h, respectively (Fig. 7e). Both findings indicated that SPS could inhibit the migration of HUVECs markedly. Notably, the repression of HUVECs migration induced by SPS occurred at exposure times and concentrations where cell viability was not obviously affected, suggesting that SPS indeed exerted its anti-angiogenic effect by inhibiting endothelial cell migration.

### SPS disrupted the tube formation but not the preformed vascular network of HUVECs

Considering that SPS dramatically inhibited HUVECs proliferation and motility, we further explored the effects of SPS on capillary-like tube structure of HUVECs, which is crucial to angiogenesis. The three-dimensional layer of Matrigel experiment was conducted and the data were shown in Fig. 7f. After incubation for 6 h, new capillary-like tube structure was clearly found in the untreated cells (Fig. 7f, panel I), while the new capillary-like tube structure formation was inhibited significantly by SPS treatment (Fig. 7f, panels II and III). Moreover, higher concentration of SPS (0.6 mg/ml) abrogated the cord formation completely (Fig. 7f, panel IV). These results demonstrated that SPS repressed endothelial cell tube formation.

In the preformed vasculature disrupting experiment, different concentrations of SPS (0.2, 0.4 and 0.6 mg/ml) were added after vascular network had already formed on Matrigel for 2 h, then the disruption of SPS on the preformed tubes was examined. The results showed that SPS treatment could not affect the preformed vascular network as it did on the tube formation as described above (Fig. 7g, panels I, II, III and IV). Additionally, the inhibitory effect of SPS on the new cord formation occurred before endothelial cell proliferation was dramatically affected (Fig. 7a), indicating that SPS indeed prevented the process of angiogenesis by inhibiting the tubular structure formation but not disrupting the preformed capillary tubes.

### SPS decreased VEGF and VEGFR expressions in HUVECs

VEGF and its receptor (VEGFR) are important regulators of angiogenesis. To detect the expressions of VEGF and VEGFR in HUVECs treated with different concentrations of SPS, western blot analysis was performed. As shown in Fig. 7h, the protein levels of VEGF and VEGFR reduced significantly in HUVECs when treated with certain dose of SPS, and the reduction was in a concentration-dependent relationship (Fig. 7i). These results suggested that inhibiting the protein expressions of VEGF and VEGFR might contribute to the anti-angiogenic activity of SPS in HUVECs.

Altogether, these data suggested that SPS possessed potent anti-angiogenic activity on HUVECs *in vitro* via inhibiting the protein expressions of VEGF and VEGFR.

### SPS blocked vessel formation in Zebrafish embryos *in vivo*

**Figure 8:**
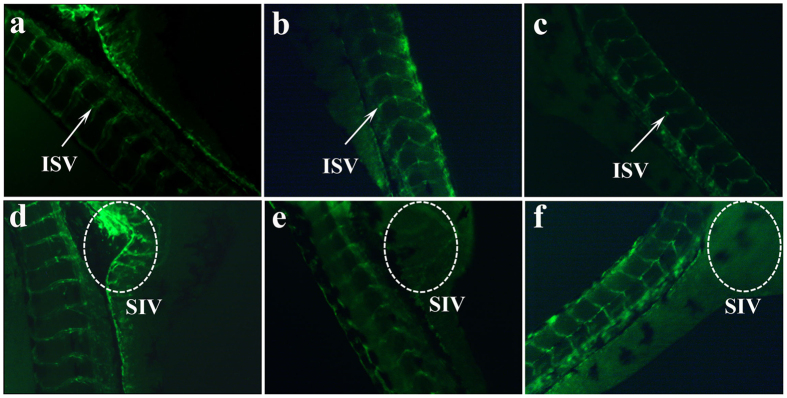
SPS affected vessel formation in zebrafish embryos. *Tg (fli1a:EGFP) y1* transgenic zebrafish embryos (n = 30) were treated without (**a**,**d**) or with 1.0 (**b**,**e**) and 4.0 (**c**,**f**) mg/ml of SPS for 48 h, then embryos were observed for viability and gross morphological changes under a fluorescence microscope. Intersegmental vessel (ISV) and basket-like structure subintestinal vessel (SIV) were indicated by white arrows and dotted line circles, respectively.

Zebrafish is an excellent animal model for the study of angiogenesis. In the present study, we further confirmed the anti-angiogenic activity of SPS *in vivo* using *Tg (fli1a:EGFP) y1* transgenic zebrafish, which expresses enhanced green fluorescent protein (EGFP) in its endothelial cells of the vasculature, thus providing more resolution and more definitive results. Intersegmental vessels (ISVs) and subintestinal vessels (SIVs) are the most readily observed angiogenic vessels in zebrafish embryos at 72 hpf, and both of them were examined by a fluorescence microscope in our study after zebrafish embryos were treated with various concentrations of SPS for 48 h. As shown in Fig. 8, SPS could dose-dependently inhibit the growth of ISVs and SIVs. Zebrafish in control group formed normal and mature ISVs (Fig. 8a) and developed a smooth basket-like structure SIVs (Fig. 8d). However, ISV formation and SIV basket growth of zebrafish embryos were markedly inhibited by SPS; some of ISVs and SIVs were completely absent, while others were incompletely formed (Fig. 8b,c,e,f), resulting in a significant reduction in the total number of mature ISVs and SIVs compared with the control. The results suggested that SPS impaired angiogenesis both *in vitro* and *in vivo*.

## Discussion

In spite of great advances in the treatment of lung carcinogenesis, the growing incidence of the disease and the severely limited efficacy have spurred efforts to develop novel and effective treatment approaches. Nowadays, the medical substances from herbal or natural sources, rather than synthetic drugs, are becoming more popular and provide alternative treatment options for patients^38^. In the present study, we evaluated the antitumor and anti-angiogenic properties of SPS, a novel polysaccharide isolated from *Sargassum integerrimum*. SPS inhibited the proliferation and induced mitochondria-mediated intrinsic apoptosis and cell cycle arrest in lung cancer cells. Besides, SPS exerted its anti-angiogenic activity both on HUVECs *in vitro* and zebrafish embryos *in vivo*.

Inhibition of cancer cell proliferation is a critical effect of anticancer agents. The induction of apoptosis, one of the principle mechanisms by which anti-carcinogenic drugs killed cancer cells, can account for the cancer cell growth inhibition. In this study, the novel polysaccharide SPS markedly repressed the proliferation of cancer cells, and induced the mitochondria-mediated intrinsic apoptosis in A549 lung cancer cells, as verified by loss of mitochondrial membrane potential, generation of intracellular reactive oxygen species, an increase in the ratio of Bax/Bcl-2 and the activation of cleaved caspase-3, caspase-9 and PARP.

Cell cycle arrest, another major target for cancer therapy, also can contribute to the inhibition of cancer cells proliferation. Cell cycle distribution is generally considered as a primary parameter in cell survival, growth and proliferation^39^ and uncontrolled cell proliferation is the typical characteristic of cancer^40^. Once the normal progression of cell cycle is affected by cell stress like DNA damage, cells will especially arrest in G2/M or S phase^41^. If DNA damage is not repaired, cells will stop the aberrant cell division^42^ and undergo apoptosis or necrosis. In the present research, the G2/M phase cell cycle arrest, together with the mitochondria-mediated intrinsic apoptosis are responsible for the antitumor activity of SPS against lung cancer.

Of all non-small cell lung cancer cases, 65–75% is detected as locally advanced or metastatic disease, which is highly correlated to the formation of angiogenesis. Angiogenesis is known to be a complex process containing multiple steps^43^, and SPS could disrupt these steps in endothelial cells, including the proliferation, migration and capillary-like structures formation. This is similar to the fucoidan extract derived from *Undaria pinnatifida*, which exerted anti-angiogenic activity by inhibiting the necessary processes of new vessel formation^44^. Notably, SPS displayed much stronger inhibitory effect on migration and cord formation than the proliferation of HUVECs. Moreover, SPS dramatically affected the viability of HUVECs at relatively longer period and higher concentration, indicating that the inhibition on the mitogenesis of HUVECs did not contribute to the repression on migration and tube formation induced by SPS. Importantly, the selecting inhibitory effect on the formation of new vessels but not the preformed tubes, suggested that SPS probably inhibited angiogenesis selectively without any influence on the pre-existing vascular structures, which showed that SPS had the great potential to be developed as anti-angiogenic drugs with minimum side effects.

The angiogenesis-inhibiting activity of SPS was further verified using zebrafish embryo model *in vivo*. Compared with the traditional chick embryo chorioallantoic membrane model (CAM), the zebrafish embryo model possesses several remarkable advantages, including ease of maintenance, transparency of the embryos, and permitting visual observations of developing cells and organs^45^,^46^. The zebrafish experiments showed that SPS could markedly block the formation of intersegmental vessels (ISVs) and subintestinal vessels (SIVs) in zebrafish embryos, thus further confirmed its anti-angiogenic effect *in vivo*, which was consistent with the results of HUVEC system *in vitro*. Yu *et al*. reported that ethanol extract of Herba Epimedii affected angiogenesis by acting on VEGF-VEGFR and ANGPT-TIE pathways in zebrafish embryos^47^. However, for SPS, the exact mechanism of action on vasculogenesis in zebrafish embroys still need to be elucidated in the future.

Angiogenesis is tightly regulated by a large number of angiogenic factors and their receptors. Among these factors, VEGF and its receptor (VEGFR) play a crucial role in tumor-associated angiogenesis and induce tumor growth and metastasis^48^. Therefore, targeting these factors has been pursued as a therapeutic strategy for inhibition of angiogenesis and neovascular survival in tumors^49^,^50^. A number of anti-angiogenic agents which inhibit VEGF and VEGFR have been developed^48^,^51^. Our present study showed that SPS displayed good repeatability of reduction of the expressions of VEGF and VEGFR, indicating that the anti-angiogenic effect of SPS appeared to be related to its inhibition of the expressions of VEGF and VEGFR. In addition, SPS could also induce cancer cell death via mitochondria-mediated apoptotic pathway and cell cycle arrest. Collectively, SPS is potential to serve as a leading agent for the development of anticancer drugs in the future.

Notably, polysaccharides originated from marine algae have recently been found to possess multiple biological activities, including antioxidant^52^, antiviral^53^ and anticancer^54^. Our present research showed that algal polysaccharides SPS not only dispalyed anticancer activity through mitochondria-mediated intrinsic apoptosis and cell cycle arrest, but also possessed potent anti-angiogenic activity both on HUVECs *in vitro* and zebrafish embryos *in vivo*. This work will benefit the future drug development of SPS for the treatment of lung cancer and opens up a new line of studies on the anticancer and anti-angiogenic potential of algal polysaccharides.

## Materials and Methods

### Materials

Fetal bovine serum (FBS) and RPMI-1640 were purchased from GIBCO (Invitrogen, Grand Island, NY, USA). Hoechst 33258 solution, JC-1 mitochondrial membrane potential detection assay kit, reactive oxygen species assay kit and cell cycle detection kit were supplied by Beyotime Institute of Biotechnology (Shanghai, China). Annexin V-FITC/PI apoptosis detection kit was obtained from Nanjing KeyGEN Biotech. Co., Ltd. (Nanjing, Jiangsu, China). The enhanced chemiluminescence (ECL) was provided by Pierce (Thermo Scientific, New Hampshire, USA). The antibodies against P53, Bax, Bcl-2, VEGF, VEGFR, GADPH and β-actin were purchased from Wuhan Boster Biological Technology, Co., Ltd. (Wuhan, Hubei, China). Antibodies against PARP, cleaved-PARP, Caspase-3, cleaved- Caspase-3, Caspase-9 and cleaved-Caspase-9 were obtained from Cell Signaling Technology (Beverly, MA, USA). Transwell inserts with 8 μm pores polycarbonate filters were provided by Corning company (Corning Costar, Cambridge, MA, USA). Matrigel was the product of BD company (Becton Dickinson, Bedford, MA, USA).

### Cell culture

Human lung cancer cell line (A549) and human umbilical vein endothelial cells (HUVECs) were purchased from American Type Culture Collection and cultured at 37 °C in a humidified atmosphere of 5% CO_2_ and 95% air in RPMI-1640 supplemented with 10% FBS.

### Cell proliferation viability assay

Viabilities of A549 and HUVECs were measured by MTT assay. Briefly, logarithmically growing cells were trypsinized from culture dishes and placed into 96-well plate and cultured at 37 °C for 24 h. Cells were treated with the varying concentrations of SPS (0, 0.2, 0.3, 0.4, 0.5, 0.6, 0.7, 0.8, 0.9, 1.0 and 1.5 mg/ml) for 12 and 24 h, and then 20 μl MTT solution (5 mg/ml) was added into each well. After incubated for 4 h, medium was removed and 150 μl of DMSO was added to each well to dissolve purple crystals of formazan with shaking at 260 rpm for 10 min. Absorbance was measured at 490 nm by a multi-detection microplate reader (infinite M1000 Pro, TECAN, Mannedorf, Switzerland). Relative cell viability was presented as a percentage relative to the control group. The 50% inhibitory concentration (IC_50_) value was determined as the concentration that caused 50% inhibition of cell proliferation. All experiments were performed three times.

### Hoechst 33258 staining

A549 cells were seeded into 12-well plates, followed by incubation with SPS (0, 0.2, 0.4 and 0.6 mg/ml) for 12 h. Then the cells were fixed with 4% paraformaldehyde for 10 min, washed three times with PBS and stained with Hoechst 33258 solution for another 10 min at room temperature in the dark. Finally, the cells were observed under fluorescence microscopy (Imager A2, Zeiss, Oberkochen, Baden-Württemberg, Germany).

### Flow cytometric analysis of apoptosis

Detection of SPS-induced apoptosis was performed by flow cytometry (FACS Aria^TM^ II, BD, San Jose, California, USA) using a commercially available Annexin V-FITC/PI apoptosis detection kit. A549 cells (1 × 10^6^) were seeded into 6-well plate and treated with serial concentrations of SPS (0, 0.4, 0.8 and 1.0 mg/ml) for 12 h. The treated cells were harvested and washed with PBS twice. The cell pellets were resuspended in 500 μl of binding buffer, followed by added 5 μl of Annexin V-FITC and PI. After incubated for 10 min at room temperature in the dark, stained cells were analyzed by flow cytometry.

### Analysis of the mitochondrial membrane potential (MMP)

A membrane-permeable lipophilic cationic fluorescent carbocyanine dye, JC-1, was often used to probe the changes of MMP during the early stages of apoptosis. In brief, A549 cells were seeded in 6-well plate and incubated in the absence or presence of SPS (0, 0.4, 0.8 and 1.0 mg/ml) for 12 h, and the cells were incubated further with JC-1 for 30 min at 37 °C in the complete medium. After centrifuged, the cell pellets were washed with staining buffer twice and resuspended in the assay buffer before detected by flow cytometry. Aggregates and monomers of JC-1 were measured in the FL2 channel (red fluorescence) and FL1 channel (green fluorescence), respectively.

### Reactive oxygen species (ROS) measurement

The generation of ROS was determined with 2′, 7′-dichlorofluorescein diacetate (DCFH-DA). In brief, A549 cells were cultured for 12 h with the medium containing different concentrations of SPS (0, 0.4, 0.8 and 1.0 mg/ml). After the culture was removed, cells were incubated with DCFH-DA in serum free medium at 37 °C for 20 min and washed three times with serum free medium. Then the cells were collected and further analyzed by flow cytometry.

### Cell cycle analysis

Cell cycle distribution was studied by measuring the DNA content of nuclei labeled with propidium iodide (PI). A549 cells were treated with varying concentrations of SPS for 24 h. After treatment, cells were harvested by centrifugation, washed with ice-cold PBS and fixed in 70% cold ethanol at 4 °C for 12 h. Thereafter, cells were washed twice and stained with Rnase (10 μg/ml) and PI (50 μg/ml) for 30 min at 37 °C in the dark. Cell cycle distribution was performed using flow cytometry and the percentages of cells at G1, S and G2/M phases were calculated.

### Western blot analysis

Western blot was conducted to detect the expressions of proteins related to apoptosis and angiogenesis. In short, A549 and HUVECs were treated with various concentrations of SPS for 24 h, and cells were collected by centrifugation, counted using a haemacytometer and lysed with sample lysis buffer (Sigma-Aldrich, Saint Louis, Missouri, USA). After that, protein samples were resolved on 10% SDS-PAGE gels, electro-transferred to nitrocellulose membranes and incubated with primary antibodies and secondary antibodies, and finally detected by enhanced chemiluminescence. The primary anti-P53, anti-Bax, anti-Bcl-2, anti-PARP, anti-Caspase-3, anti-Caspase-9, anti-cleaved-PARP, anti-cleaved-Caspase -3, anti-cleaved-Caspase-9, anti-VEGF and anti-VEGFR antibodies were used for this study. Anti-β-actin and anti-GADPH antibodies were used to normalize for protein loading.

### Scratch-wound migration assay

Scratch-wound migration assay was conducted to assess the migration of HUVECs. HUVECs were incubated in serum-free medium overnight for synchronization and then trypsinized from culture dishes and placed into 96-well plate. After growth to 90% confluence, the scratch-wounds were made by scraping the cell monolayers with sterile pipet tips. Washed twice with PBS, the cells were exposed to medium supplemented with 2% FBS with or without SPS (0, 0.2, 0.3 and 0.4 mg/ml). After incubation for 10 h, three fields of each wound were selected and photographed with an inverted microscope (NIKON TS100, Tokyo, Japan) equipped with a digital camera. The cell migration rate was quantified by measuring the ratio of migration distance to total distance of the wound gap.

### Transwell migration assay

Transwell Boyden chamber was used to determine the migration of HUVECs. Briefly, the lower compartment contained 0.6 ml of RPMI-1640 medium supplemented with 20% FBS. HUVEC cells (5 × 10^5^) were resuspended in 100 μl medium containing 1% FBS and different concentrations of SPS (0, 0.2, 0.3 and 0.4 mg/ml), and seeded into the upper compartment of each well. Cells were then incubated for 8 h to allow cell migration through the filter membrane to the lower side of the insert. After washed with PBS, cells were fixed with 95% ethanol and stained with 0.1% crystal violet. Then the non-migrated cells on the upper side of the filter were gently removed using cotton swabs and the migrated cells on the lower side of the filter were observed and counted in five random fields. The inhibition rate of migration was calculated as follows: Inhibition rate = [1 − (migrated cells treated/migrated cells control)] × 100%.

### Tube formation assay

The effect of SPS on the ability of HUVECs to form capillary-like structures on Matrigel was evaluated. Briefly, Matrigel which was thawed at 4 °C overnight, was diluted with serum-free medium, layered in a 96-well plate and incubated at 37 °C for 30 min to allow polymerization. Then, HUVECs (3 × 10^4^ cell/well) were plated onto the Matrigel layer in the culture medium containing varying concentrations of SPS (0, 0.2, 0.4 and 0.6 mg/ml). After incubation for 6 h, the tube-like structure was visualized and imaged under an inverted microscope (NIKON TS100, Tokyo, Japan). For the preformed vasculature disrupting experiments, we conducted it according to the method described before with minor modification^44^. In short, the certain concentrations of SPS (0, 0.2, 0.4 and 0.6 mg/ml) were added to the cells after tube formation had come into being for 2 h. After SPS administration for 12 h, the disruption on the tube-like structure was photographed from randomly chosen fields by an inverted microscope.

### Zebrafish culture, embryo handling and drug administration

*Tg* (*fli1a:EGFP*) *y1* transgenic zebrafish were maintained as described before^55^ and all zebrafish experiments in this study were approved by IOCAS (Institute of Oceanology, Chinese Academy of Sciences) Laboratory Animal Care and Ethics Committee in accordance with the animal care and use guidelines. *Tg* (*fli1a:EGFP*) *y1* transgenic zebrafish were maintained at 28 °C on a 14/10 h (light/dark) photoperiod and food was freely available. The zebrafish embryos were generated by natural pair-wise mating (3–12 months old). Dead or unfertilized embryos were removed in the first few hours of development and healthy embryos were selected and arrayed in 24-well plates with ten embryos per well. Embryos at 24 h post-fertilization (hpf) were treated with 1 and 4mg/ml of SPS for 48 h, with the corresponding solvent served as vehicle control. Each sample group contained 30 embryos. After drug administration, the enhanced green fluorescent proteins-expressing endothelial cells of the vasculature in intersegmental blood vessels (ISVs) and subintestinal vessel plexus (SIVs) were observed and recorded using a fluorescence microscopy.

### Statistical analysis

All data were expressed as means ± SD. Statistical analysis was performed using student’s *t*-test to determine the significance between groups. Differences of *P *< 0.05 were considered statistically significant.

## Additional Information

**How to cite this article**: Liu, G. *et al*. A novel polysaccharide from *Sargassum integerrimum* induces apoptosis in A549 cells and prevents angiogensis *in vitro* and *in vivo. Sci. Rep.* **6**, 26722; doi: 10.1038/srep26722 (2016).

## Supplementary Material

Supplementary Information

## References

[b1] Kumar R. . A phase 1b trial of the combination of an all-oral regimen of capecitabine and erlotinib in advanced non-small cell lung cancer in Caucasian patients. Cancer Chemother. Pharmacol. 77, 375–383 (2016). 26706729 10.1007/s00280-015-2950-1

[b2] Punathil T. & Katiyar S. K. Inhibition of non-small cell lung cancer cell migration by grape seed proanthocyanidins is mediated through the inhibition of nitric oxide, guanylate cyclase, and ERK1/2. Mol. Carcinog. 48, 232–242 (2009). 18680102 10.1002/mc.20473

[b3] Lowe S. W. & Lin A. W. Apoptosis in cancer. Carcinogenesis 21, 485–495 (2000). 10688869 10.1093/carcin/21.3.485

[b4] Chen G. . Polysaccharides from Rhizopus nigricans mycelia induced apoptosis and G2/M arrest in BGC-823 cells. Carbohydr. Polym. 97, 800–808 (2013). 23911518 10.1016/j.carbpol.2013.05.068

[b5] Fesik S. W. Promoting apoptosis as a strategy for cancer drug discovery. Nat. Rev. Cancer 5, 876–885 (2005). 16239906 10.1038/nrc1736

[b6] Ellis L. M. . Overview of angiogenesis: Biologic implications for antiangiogenic therapy. Semin. Oncol. 28, 94–104 (2001). 11706401 10.1016/s0093-7754(01)90287-8

[b7] Folkman J. Tumor angiogenesis: therapeutic implications. N. Engl. J. Med. 285, 1182–1186 (1971). 4938153 10.1056/NEJM197111182852108

[b8] O’Reilly M. S. The preclinical evaluation of angiogenesis inhibitors. Invest. New Drugs 15, 5–13 (1997). 9195285 10.1023/a:1005762410476

[b9] Chan L. S., Daruwalla J. & Christophi C. Selective targeting of the tumour vasculature. ANZ. J. Surg. 78, 955–967 (2008). 18959693 10.1111/j.1445-2197.2008.04712.x

[b10] Deplanque G. & Harris A. L. Anti-angiogenic agents: clinical trial design and therapies in development. Eur. J. Cancer 36, 1713–1724 (2000). 10959057 10.1016/s0959-8049(00)00149-0

[b11] Loges S., Schmidt T. & Carmeliet P. Mechanisms of resistance to anti-angiogenic therapy and development of third-generation anti-angiogenic drug candidates. Genes Cancer 1, 12–25 (2010). 21779425 10.1177/1947601909356574 PMC3092176

[b12] Zong A. . Anti-tumor activity and the mechanism of SIP-S: A sulfated polysaccharide with anti-metastatic effect. Carbohydr. Polym. 129, 50–54 (2015). 26050887 10.1016/j.carbpol.2015.04.017

[b13] Zong A., Cao H. & Wang F. Anticancer polysaccharides from natural resources: a review of recent research. Carbohydr. Polym. 90, 1395–1410 (2012). 22944395 10.1016/j.carbpol.2012.07.026

[b14] Chowdhury S. R. . Low fucose containing bacterial polysaccharide facilitate mitochondria-dependent ROS-induced apoptosis of human lung epithelial carcinoma via controlled regulation of MAPKs-mediated Nrf2/Keap1 homeostasis signaling. Mol. Carcinog. 54, 1636–1655 (2015). 25358602 10.1002/mc.22236

[b15] Zhang C. . Grateloupia longifolia polysaccharide inhibits angiogenesis by downregulating tissue factor expression in HMEC-1 endothelial cells. Br. J. Pharmacol. 148, 741–751 (2006). 16715123 10.1038/sj.bjp.0706741 PMC1617078

[b16] Koyanagi S., Tanigawa N., Nakagawa H., Soeda S. & Shimeno H. Oversulfation of fucoidan enhances its anti-angiogenic and antitumor activities. Biochem. Pharmacol. 65, 173–179 (2003). 12504793 10.1016/s0006-2952(02)01478-8

[b17] Matsubara K., Mori M., Matsumoto H., Hori K. & Miyazawa K. Antiangiogenic properties of a sulfated galactan isolated from a marine alga, Codium cylindricum. J. Appl. Phycol. 15, 87–90 (2002).

[b18] Park M. H., Nam Y. H. & Han J. S. Sargassum coreanum extract alleviates hyperglycemia and improves insulin resistance in db/db diabetic mice. Nutr. Res. Pract. 9, 472–479 (2015). 26425276 10.4162/nrp.2015.9.5.472 PMC4575959

[b19] Chater P. I., Wilcox M. D., Houghton D. & Pearson J. P. The role of seaweed bioactives in the control of digestion: implications for obesity treatments. Food Funct. 6, 3420–3427 (2015). 26416783 10.1039/c5fo00293a

[b20] Jin W. . A study of neuroprotective and antioxidant activities of heteropolysaccharides from six Sargassum species. Int. J. Biol. Macromol. 67, 336–342 (2014). 24680812 10.1016/j.ijbiomac.2014.03.031

[b21] Kim S. H. . Antioxidant Activity of Sulfated Polysaccharides Isolated from Sargassum fulvellum. J. Food Sci Nutr. 12, 65–73 (2007).

[b22] Ye H., Wang K., Zhou C., Liu J. & Zeng X. Purification, antitumor and antioxidant activities *in vitro* of polysaccharides from the brown seaweed Sargassum pallidum. Food Chem. 111, 428–432 (2008). 26047446 10.1016/j.foodchem.2008.04.012

[b23] Gil J., Almeida S., Oliveira C. R. & Rego A. C. Cytosolic and mitochondrial ROS in staurosporine-induced retinal cell apoptosis. Free Radic. Biol. Med. 35, 1500–1514 (2003). 14642398 10.1016/j.freeradbiomed.2003.08.022

[b24] Zamzami N. . Reduction in mitochondrial potential constitutes an early irreversible step of programmed lymphocyte death *in vivo*. J. Exp. Med. 181, 1661–1672 (1995). 7722446 10.1084/jem.181.5.1661 PMC2192017

[b25] Barbu A., Welsh N. & Saldeen J. Cytokine-induced apoptosis and necrosis are preceded by disruption of the mitochondrial membrane potential (Deltapsi(m)) in pancreatic RINm5F cells: prevention by Bcl-2. Mol. Cell. Endocrinol. 190, 75–82 (2002). 11997180 10.1016/s0303-7207(02)00009-6

[b26] Yang L. . Fucoidan derived from Undaria pinnatifida induces apoptosis in human hepatocellular carcinoma SMMC-7721 cells via the ROS-mediated mitochondrial pathway. Mar. Drugs. 11, 1961–1976 (2013). 23752353 10.3390/md11061961 PMC3721216

[b27] Turrens J. F. Superoxide production by the mitochondrial respiratory chain. Biosci. Rep. 17, 3–8 (1997). 9171915 10.1023/a:1027374931887

[b28] Park J., Lee J. & Choi C. Mitochondrial network determines intracellular ROS dynamics and sensitivity to oxidative stress through switching inter-mitochondrial messengers. PLoS One 6, e23211; doi: 10.1371/journal.pone.0023211 (2011). 21829717 PMC3150422

[b29] Wu T. S., Liao Y. C., Yu F. Y., Chang C. H. & Liu B. H. Mechanism of patulin-induced apoptosis in human leukemia cells (HL-60). Toxicol. Lett. 183, 105–111 (2008). 18992795 10.1016/j.toxlet.2008.09.018

[b30] Lowe S. W., Schmitt E. M., Smith S. W., Osborne B. A. & Jacks T. p53 is required for radiation-induced apoptosis in mouse thymocytes. Nature 362, 847–849 (1993). 8479522 10.1038/362847a0

[b31] Haunstetter A. & Izumo S. Apoptosis: basic mechanisms and implications for cardiovascular disease. Circ. Res. 82, 1111–1129 (1998). 9633912 10.1161/01.res.82.11.1111

[b32] Burlacu A. Regulation of apoptosis by Bcl-2 family proteins. J. Cell. Mol. Med. 7, 249–257 (2003). 14594549 10.1111/j.1582-4934.2003.tb00225.x PMC6741335

[b33] Autret A. & Martin S. J. Emerging role for members of the Bcl-2 family in mitochondrial morphogenesis. Mol. Cell 36, 355–363 (2009). 19917245 10.1016/j.molcel.2009.10.011

[b34] Gross A., McDonnell J. M. & Korsmeyer S. J. BCL-2 family members and the mitochondria in apoptosis. Genes Dev. 13, 1899–1911 (1999). 10444588 10.1101/gad.13.15.1899

[b35] Acehan D. . Three-dimensional structure of the apoptosome: implications for assembly, procaspase-9 binding, and activation. Mol. Cell 9, 423–432 (2002). 11864614 10.1016/s1097-2765(02)00442-2

[b36] Pradelli L. A., Beneteau M. & Ricci J. E. Mitochondrial control of caspase-dependent and -independent cell death. Cell. Mol. Life Sci. 67, 1589–1597 (2010). 20151314 10.1007/s00018-010-0285-y PMC11115767

[b37] Yakovlev A. G. . A role of the Ca^2+^/Mg^2+^-dependent endonuclease in apoptosis and its inhibition by Poly(ADP-ribose) polymerase. J. Biol. Chem. 275, 21302–21308 (2000). 10807908 10.1074/jbc.M001087200

[b38] Kim K. M. . 5,3′-Dihydroxy-6,7,4′-trimethoxyflavanone exerts its anticancer and antiangiogenesis effects through regulation of the Akt/mTOR signaling pathway in human lung cancer cells. Chem. Biol. Interact. 225, 32–39 (2015). 25446852 10.1016/j.cbi.2014.10.033

[b39] Cheng F. . A Natural Triterpene Derivative from Euphorbia kansui Inhibits Cell Proliferation and Induces Apoptosis against Rat Intestinal Epithelioid Cell Line *in Vitro*. Int. J. Mol. Sci. 16, 18956–18975 (2015). 26274958 10.3390/ijms160818956 PMC4581281

[b40] Hanahan D. & Weinberg R. A. Hallmarks of cancer: the next generation. Cell 144, 646–674 (2011). 21376230 10.1016/j.cell.2011.02.013

[b41] Abid-Essefi S. . DNA fragmentation, apoptosis and cell cycle arrest induced by zearalenone in cultured DOK, Vero and Caco-2 cells: prevention by Vitamin E. Toxicology 192, 237–248 (2003). 14580790 10.1016/s0300-483x(03)00329-9

[b42] Bjelland S. & Seeberg E. Mutagenicity, toxicity and repair of DNA base damage induced by oxidation. Mutat. Res. 531, 37–80 (2003). 14637246 10.1016/j.mrfmmm.2003.07.002

[b43] Patan S. Vasculogenesis and angiogenesis as mechanisms of vascular network formation, growth and remodeling. J. Neurooncol. 50, 1–15 (2000). 11245270 10.1023/a:1006493130855

[b44] Liu F. . Fucoidan extract derived from Undaria pinnatifida inhibits angiogenesis by human umbilical vein endothelial cells. Phytomedicine 19, 797–803 (2012). 22510492 10.1016/j.phymed.2012.03.015

[b45] Serbedzija G. N., Flynn E. & Willett C. E. Zebrafish angiogenesis: a new model for drug screening. Angiogenesis 3, 353–359 (1999). 14517415 10.1023/a:1026598300052

[b46] Parng C., Seng W. L., Semino C. & McGrath P. Zebrafish: a preclinical model for drug screening. Assay Drug Dev. Technol. 1, 41–48 (2002). 15090155 10.1089/154065802761001293

[b47] Yu X. B. . Anti-Angiogenic Activity of Herba Epimedii on Zebrafish Embryos *In vivo* and HUVECs *In Vitro*. Phytotherapy Research 27, 1368–1375 (2013). 23147754 10.1002/ptr.4881

[b48] Liang X. . VEGF signal system: the application of antiangiogenesis. Curr. Med. Chem. 21, 894–910 (2014). 24059233 10.2174/09298673113206660264

[b49] Suzuki Y., Montagne K., Nishihara A., Watabe T. & Miyazono K. BMPs promote proliferation and migration of endothelial cells via stimulation of VEGF-A/VEGFR2 and angiopoietin-1/Tie2 signalling. J. Biochem. 143, 199–206 (2008). 18006519 10.1093/jb/mvm215

[b50] Kiselyov A., Balakin K. V. & Tkachenko S. E. VEGF/VEGFR signalling as a target for inhibiting angiogenesis. Expert. Opin. Investig. Drugs 16, 83–107 (2007). 10.1517/13543784.16.1.83 17155856

[b51] Murphy D. A. . Inhibition of tumor endothelial ERK activation, angiogenesis, and tumor growth by sorafenib (BAY43-9006). Am. J. Pathol. 169, 1875–1885 (2006). 17071608 10.2353/ajpath.2006.050711 PMC1780219

[b52] Wang H. . An overview on natural polysaccharides with antioxidant properties. Curr. Med. Chem. 20, 2899–2913 (2013). 23627941 10.2174/0929867311320230006

[b53] Wang W., Wang S. X. & Guan H. S. The antiviral activities and mechanisms of marine polysaccharides: an overview. Mar. Drugs. 10, 2795–2816 (2012). 23235364 10.3390/md10122795 PMC3528127

[b54] Zorofchian Moghadamtousi S. . Anticancer and antitumor potential of fucoidan and fucoxanthin, two main metabolites isolated from brown algae. The Scientific World Journal 2014, 768323; doi: 10.1155/2014/768323 (2014). 24526922 PMC3910333

[b55] Westerfield M. The zebrafish book : a guide for the laboratory use of zebrafish (Danio rerio). (M. Westerfield, 2007).

