# Supplementary material for: A novel polysaccharide from Sargassum integerrimum induces apoptosis in A549 cells and prevents angiogensis in vitro and in vivo
Source: Sci Rep. 2016 May 24;6:26722. doi: 10.1038/srep26722 (PMC4877640; doi:10.1038/srep26722)
Supplement: Supplementary Information [file srep26722-s1.doc]

**A novel polysaccharide from *Sargassum integerrimum* induces apoptosis in A549 cells and prevents angiogensis *in vitro* and *in vivo***

Ge Liu^1, 2, 3^, Shan Kuang^1, 2^, Shimei Wu^4^, Weihua Jin^1, 2*^, Chaomin Sun^1, 2*^

^1^Key Laboratory of Experimental Marine Biology, Institute of Oceanology, Chinese Academy of Sciences, Qingdao, 266071, China

^2^Laboratory for Marine Biology and Biotechnology, Qingdao National Laboratory for Marine Science and Technology, Qingdao, 266071, China

^3^College of Earth Science, University of Chinese Academy of Sciences, Beijing, 100049, China

**^4^Key Laboratory of Biobased Materials,** Qingdao Institute of Bioenergy and Bioprocess Technology, **Chinese Academy of Sciences, Qingdao,** 266101, China

^*^Corresponding authors

Chaomin Sun Tel.: +86 532 82898857; fax: +86 532 82898648.

E-mail address: [sunchaomin@qdio.ac.cn](mailto:sunchaomin@qdio.ac.cn)

Weihua Jin Tel.: +86 532 82898703; fax: +86 532 82898703.

E-mail address: [jinweihua@qdio.ac.cn](mailto:jinweihua@qdio.ac.cn)

**Supplementary information**

**Materials and methods**

**Preparation of polysaccharide from *Sargassum integerrimum*.** *Sargassum integerrimum* was collected on June 17, 2014 in Zhanjiang, Guangdong Province, China. The fresh seaweed was washed with seawater and sun-dried. For preparation of polysaccharide, the algae (100 g) were cut into pieces and extracted with 2.5 L water at 110 °C for 4 h. The extracted solution was filtered with celite and concentrated. Further elimination of alginic acid was achieved using 20% ethanol with MgCl_2_ (0.05 M). After the alginic acid was removed, the supernatant fluid was collected, dialyzed and concentrated. Finally, after ethanol precipitation, a purified polysaccharide was obtained and named as SPS.

**Characterization of SPS.** The molar ratios of monosaccharides and the content of fucose were analyzed following the methods described previously[^1^](#_ENREF_1). Briefly, SPS (10 mg/ml) was hydrolyzed by trifluoroacetic acid (2 M) under nitrogen atmosphere for 4 h at 110 °C, and the hydrolyzed mixture was neutralized to pH 7.0 with sodium hydroxide, then the obtained monosaccharides were converted into its 1-phenyl-3-methyl-5-pyrazolone derivatives and separated by HPLC chromatography on YMC Pack ODS AQ column (4.6 × 250 mm). The results showed that SPS consisted of mannose, rhamnose, glucuronic acid, glucose, galactose, xylose and fucose in a molar ratio of 0.25:0.06:0.19:0.19:0.58:0.17:1. The sulfate content was analyzed by ion chromatography on Shodex IC SI-52 4E column (4.0 × 250 mm) eluted with 3.6 mM Na_2_CO_3_ at a flow rate of 0.8 ml/min at 45 °C, and the results indicated that sulfate content was 37.26%. Uronic acid (UA) was determined by a modified carbazole method and the content of UA was 4.88%.

The molecular weight of the polysaccharide was evaluated by GPC-HPLC on a TSK G3000 PWxl column (7 μm 7.8 × 300 mm) with elution in 0.05 M Na_2_SO_4_ at a flow rate of 0.5 ml/min at 40 °C with refractive index detection. Ten different molecular weight dextrans purchased from the National Institute for the Control of Pharmaceutical and Biological Products (China) were used as molecular weight standards. And the result showed that the average molecular weight of SPS was 122.9 kDa.

In addition, the Fourier transform infrared spectrum of SPS was also conducted and the data showed that IR spectrum of SPS displayed the characteristic peaks of polysaccharide.

**Cell cycle analysis.** Logarithmically growing HUVECs were trypsinized from culture dishes and placed into 6-well plate and cultured at 37 °C for 24 h. Then, cells were treated with various concentrations of SPS (0, 1.0, 1.5 and 2.0 mg/ml) for another 24 h. After treatment, cells were harvested by centrifugation, washed with ice-cold PBS and fixed in 70% (v/v) cold ethanol at 4 °C for 12 h. Thereafter, the fixed cells were collected and stained with RNase and PI for 30 min at 37 °C in the dark. Cell cycle distribution was performed using flow cytometry (FACS Aria^TM^ II, BD, San Jose, California, USA), and cell cycle percentage was calculated with Modifit LT software.


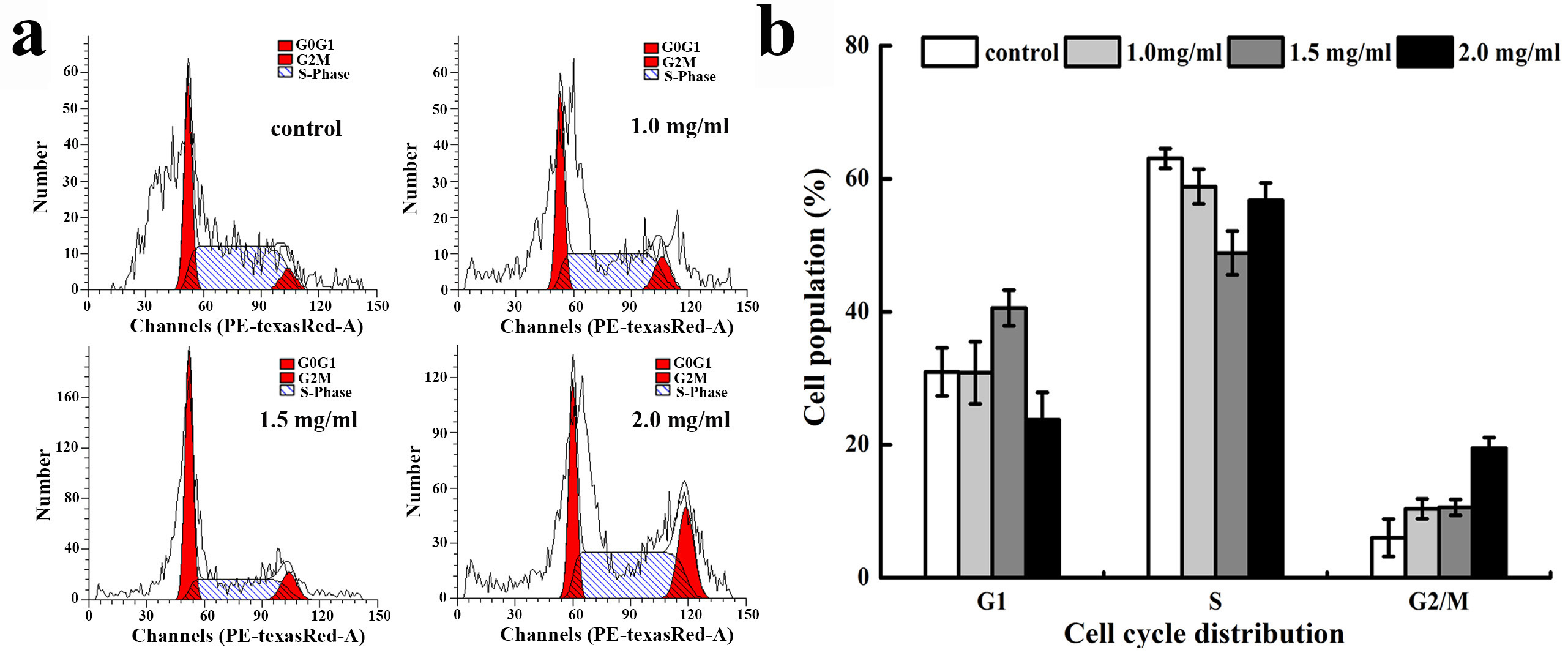


**Figure S1. SPS induced G2/M phase cell cycle arrest in HUVECs.** (**a**) Representative histograms of DNA content in the cells incubated with SPS at 0, 1.0, 1.5 and 2.0 mg/ml for 24 h and cell cycle analysis were conducted n=2. (**b**) Percentage of cell population in G1, S, and G2/M phase calculated by Modifit LT software.

**Reference**

1 Zhang, J., Zhang, Q., Wang, J., Shi, X. & Zhang, Z. Analysis of the monosaccharide composition of fucoidan by precolumn derivation HPLC*. Chinese Journal of Oceanology and Limnolo*g**y** 27, 578-582 (2009).
